# FunVFPred: Predicting fungal virulence factors using a unified representation learning model

**DOI:** 10.1016/j.isci.2026.117217

**Published:** 2026-08-13

**Authors:** Ekjot Kaur, Vishal Acharya

**Affiliations:** 1 Artificial Intelligence for Computational Biology (AICoB) Laboratory, Biotechnology Division, CSIR-Institute of Himalayan Bioresource Technology (CSIR-IHBT), Palampur, Himachal Pradesh, India; 2 Academy of Scientific and Innovative Research (AcSIR), Ghaziabad 201002, India

**Keywords:** human-fungal virulence factors, fungal pathogenicity, virulence factors prediction, protein sequence classification, computational protein annotation, machine learning in host-pathogen interaction

## Abstract

Fungal virulence factors (VFs) are proteins that support host colonization, tissue invasion, immune modulation, and disease progression. Identifying candidate VFs across pathogenic fungi can help characterize mechanisms underlying fungal-host interactions and prioritize proteins for experimental investigation. We developed FunVFPred, a machine learning framework that uses UniRep protein sequence embeddings, and multiple classifiers to predict candidate fungal VFs. Using experimentally validated VFs from human-pathogenic *Candida* species, the random forest model achieved 73.4% accuracy and a Matthews correlation coefficient (MCC) of 0.47 in 5-fold cross-validation. Evaluation on an independent *Aspergillus fumigatus* dataset yielded 85.7% accuracy and an MCC of 0.71, demonstrating the potential for cross-species prioritization of virulence-associated proteins. FunVFPred provides an accessible computational resource for investigating fungal pathogenicity and guiding the selection of candidate proteins for downstream experimental validation.

## Highlights

• FunVFPred predicts candidate VFs in human-pathogenic fungi
• Random forest achieved the best performance, with 77.4% accuracy and MCC of 0.5509
• UniRep embeddings combined with AAC and DDE improved VF prediction
• Cross-validation and independent testing supported FunVFPred’s robustness

## Graphical abstract

**Figure:**
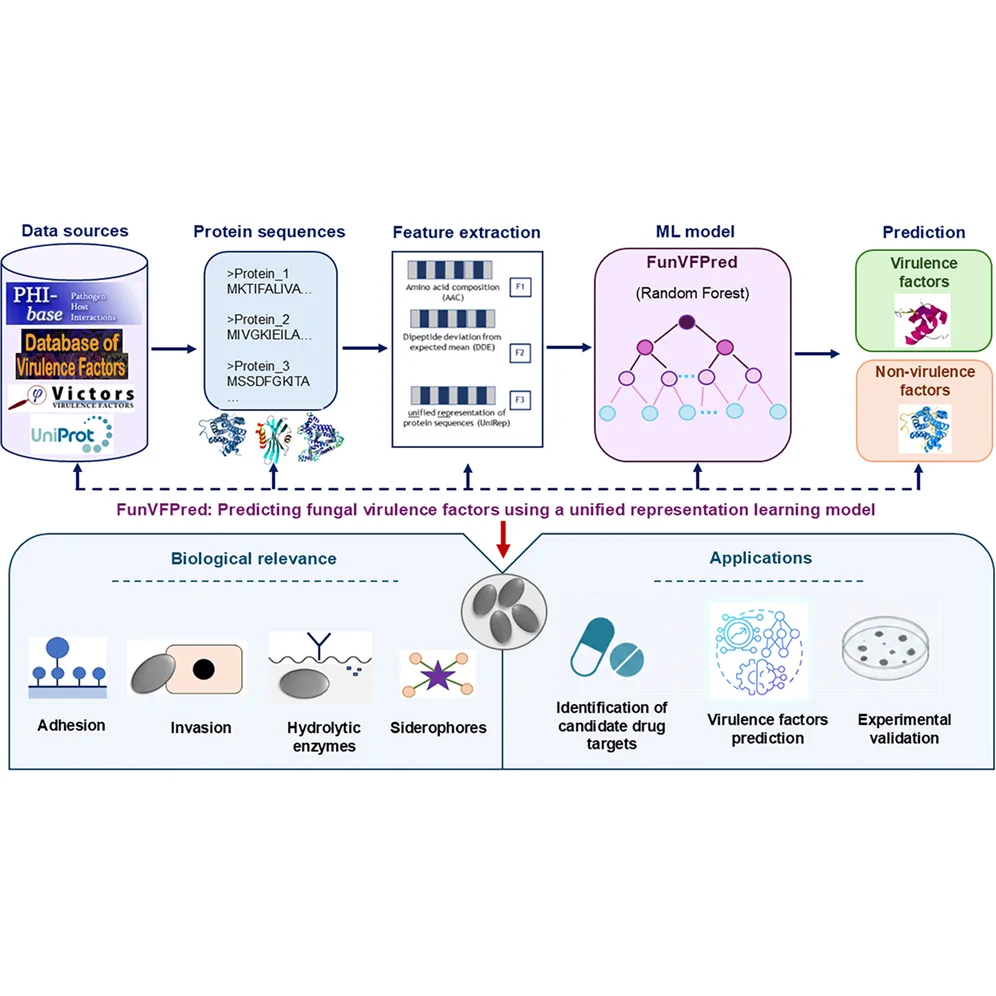

## Teaser

Applied microbiology; Biocomputational method

## Introduction

Fungi, despite being one of the three major eukaryotic kingdoms alongside animals and plants, remain comparatively underexplored. While nearly 150,000 fungal species have been identified, estimates suggest that several million remain undiscovered.^1^^,^^2^ Fungi play a vital role in sustaining life on Earth and are deeply ingrained in our cultural heritage; however, they remain comparatively neglected and poorly understood.^3^^,^^4^ Compared with bacterial and viral pathogens, fungal pathogens have historically received less attention in studies of human infectious diseases.^5^ Recent estimates suggest that fungal infections are responsible for approximately 1.6 million deaths annually worldwide.^6^ Fatal outcomes are particularly associated with allergic reactions, mucosal and superficial infections, and chronic fungal diseases. In individuals with compromised immune systems, severe fungal infections can result in mortality rates of up to 50%. Approximately 600 fungal species are known to cause human disease, including life-threatening systemic infections with high fatality rates.^7^^,^^8^ Examples include *Aspergillus fumigatus*, *Cryptococcus neoformans*, *Histoplasma capsulatum*, and *Candida albicans*.^9^^,^^10^^,^^11^ *C. albicans* is a major cause of mucosal and systemic infections worldwide.^12^ The virulence factors (VFs) that determine the ability of pathogens to cause candidiasis have a significant impact on the onset and persistence of infection. The incidence of candidiasis remains substantial, particularly in healthcare settings, despite advances in modern medicine. Even with medical intervention, mortality rates can be high.^13^^,^^14^

A critical factor determining fungal pathogenicity is the presence of VFs, specialized proteins that enable pathogens to colonize host tissues, evade immune defenses, and facilitate infection progression.^15^ These factors allow infections to persist, proliferate, and disseminate by disrupting host homeostasis and modulating immune responses. Interactions among VFs may also enhance the pathogen’s ability to adapt to the host environment. A deeper understanding of VF functions is important for developing effective antifungal strategies and improving diagnostic approaches.^16^ Virulence-associated proteins can be broadly categorized into toxins, adhesins, hydrolytic enzymes, and invasion factors that interact with host cells and the immune system to promote infection and support fungal survival and growth within the host. Pathogenic microbes possess adhesins that facilitate their attachment to host cells, helping them avoid clearance from mucosal surfaces. A distinctive feature of infectious microbes is their ability to invade host cells and tissues, which may be facilitated by filamentous growth or active penetration.

Additionally, pathogens can exploit host cells as vehicles or disrupt interepithelial connections to overcome cellular barriers.^17^ To overcome host defenses, fungi such as *C. neoformans* can employ both direct invasion and the “Trojan horse” mechanism, enabling them to bypass cellular barriers and disseminate into deeper tissues.^18^^,^^19^ Proteases and lipases are examples of hydrolytic enzymes that serve as important VFs by facilitating fungal invasion of host tissues. Even in the absence of active fungal growth, molds can release toxic secondary metabolites that contribute to tissue damage and disease progression.^20^ The production of peptide toxins by certain fungi, such as *C. albicans*, has also been documented, highlighting their potential role in pathogenesis.^21^ These toxins can be considered classical VFs because, similar to bacterial toxins, they can damage host tissues even in the absence of actively growing pathogens. In addition to toxins, effector proteins represent another important class of VFs in pathogenic microorganisms. These proteins manipulate host cellular functions to benefit the pathogen.^22^ By interacting with host molecules, effector proteins can alter intracellular signaling pathways, ultimately promoting pathogen survival and proliferation.

Effector proteins are particularly notable for their roles in manipulating host signaling and metabolism, a feature well studied in plant-pathogenic fungi such as *Ustilago maydis*.^22^^,^^23^ However, the mechanisms and targets of effector proteins in human fungal pathogens remain less well characterized. This knowledge gap limits our understanding of fungal pathogenesis and may hinder the development of targeted antifungal therapies.

While significant progress has been made in understanding bacterial and viral VFs, knowledge of fungal VFs, particularly at the molecular and protein levels, remains comparatively limited. This knowledge gap can hamper the development of effective antifungal treatments and diagnostic tools for infections caused by *Candida* species and other human fungal pathogens. Computational tools for predicting fungal VFs are also limited. Despite the availability of established methods for bacterial and viral pathogens, including BLAST and machine learning (ML) models such as support vector machines (SVMs) and neural networks,^16^^,^^24^ computational approaches for predicting fungal VFs remain relatively limited. A promising approach to addressing this challenge is the application of ML algorithms. Sachdeva et al. developed SPAAN, a neural-network-based method for predicting adhesins and adhesin-like proteins, which achieved high sensitivity in adhesin prediction.^25^ However, its application was focused primarily on adhesins. The inherent complexity and diversity of fungal genomes and proteomes present additional challenges for developing reliable predictive models, highlighting the need for specialized bioinformatics approaches.

Existing VF prediction tools, such as VirulentPred,^16^ HyperVR,^26^ and EffectorP,^27^ have demonstrated utility for predicting virulence-associated proteins or effector proteins in bacterial and plant-pathogenic systems. However, tools specifically designed to predict a broad range of VFs in human fungal pathogens remain limited. Although FungalRV^28^ was developed to predict adhesins in human-pathogenic fungi, its scope is restricted primarily to adhesins, and its application is focused largely on vaccine research. By integrating computational techniques with immunoinformatics, FungalRV facilitates the identification of potential fungal vaccine targets.

Notably, several computational approaches developed for fungal pathogens have been designed primarily for plant-fungus interaction systems. Considerable progress has been made in developing computational methods for identifying fungal effector proteins in plant-pathogenic systems. One example is EffectorP, which predicts secreted effector proteins involved in modulating plant immune responses during infection.^27^^,^^29^ Phytopathogenic fungi produce predominantly small secreted effector proteins; therefore, many of these proteins are less than 200 amino acids in length and contain a high proportion of cysteine residues, which contribute to structural stability and interactions with host plants.^29^^,^^30^ While these identification tools can be useful for studying plant-pathogenic fungi, effector proteins represent only a subset of the broader repertoire of fungal VFs.

The VFs produced by human fungal pathogens comprise an exceptionally diverse collection of proteins that enable pathogens to colonize their hosts, invade human tissues, undergo morphological transitions, and evade host immune responses. Furthermore, VFs produced by human fungal pathogens encompass not only small secreted proteins but also large adhesins, pathogenic enzymes, and regulatory proteins involved in diverse aspects of pathogenesis. For example, the *C. albicans* virulence-associated proteins ALS3 and HWP2 play important roles in establishing contact between *C. albicans* and host epithelial cells and in biofilm formation, while BUD4 and VPS11 are examples of proteins involved in morphogenesis and pathogenicity. While computational approaches for predicting effectors associated with plant-fungus interactions have been successful,^29^^,^^30^^,^^31^ these methods may not adequately capture the broader repertoire of VFs in human fungal pathogens. EffectorP and Fungtion, for example, are primarily designed to identify secreted effector proteins associated with plant-pathogen interactions.^27^^,^^31^ Consequently, their predictive models rely on features associated with plant-pathogen effector biology, including secretion signals and sequence characteristics typical of small effector proteins. In contrast, virulence determinants in human fungal pathogens encompass a more diverse range of proteins involved in adherence, host tissue invasion, metabolic adaptation to the host environment, and immune evasion. Due to the biological differences between these systems, plant effector prediction tools may not adequately capture the diversity of virulence-associated proteins involved in human fungal infections. These considerations underscore the need for computational tools specifically designed to identify virulence determinants associated with human fungal pathogenesis.

Given these challenges, there is a need for ML frameworks tailored specifically to fungal VF prediction. Such tools can aid in prioritizing candidate virulence markers and potential therapeutic targets, ultimately contributing to improved understanding and management of fungal diseases. Predicted VFs can subsequently be evaluated through domain- and function-based analyses, and their roles interpreted in the context of existing literature to assess their biological relevance.

**Figure 1:**
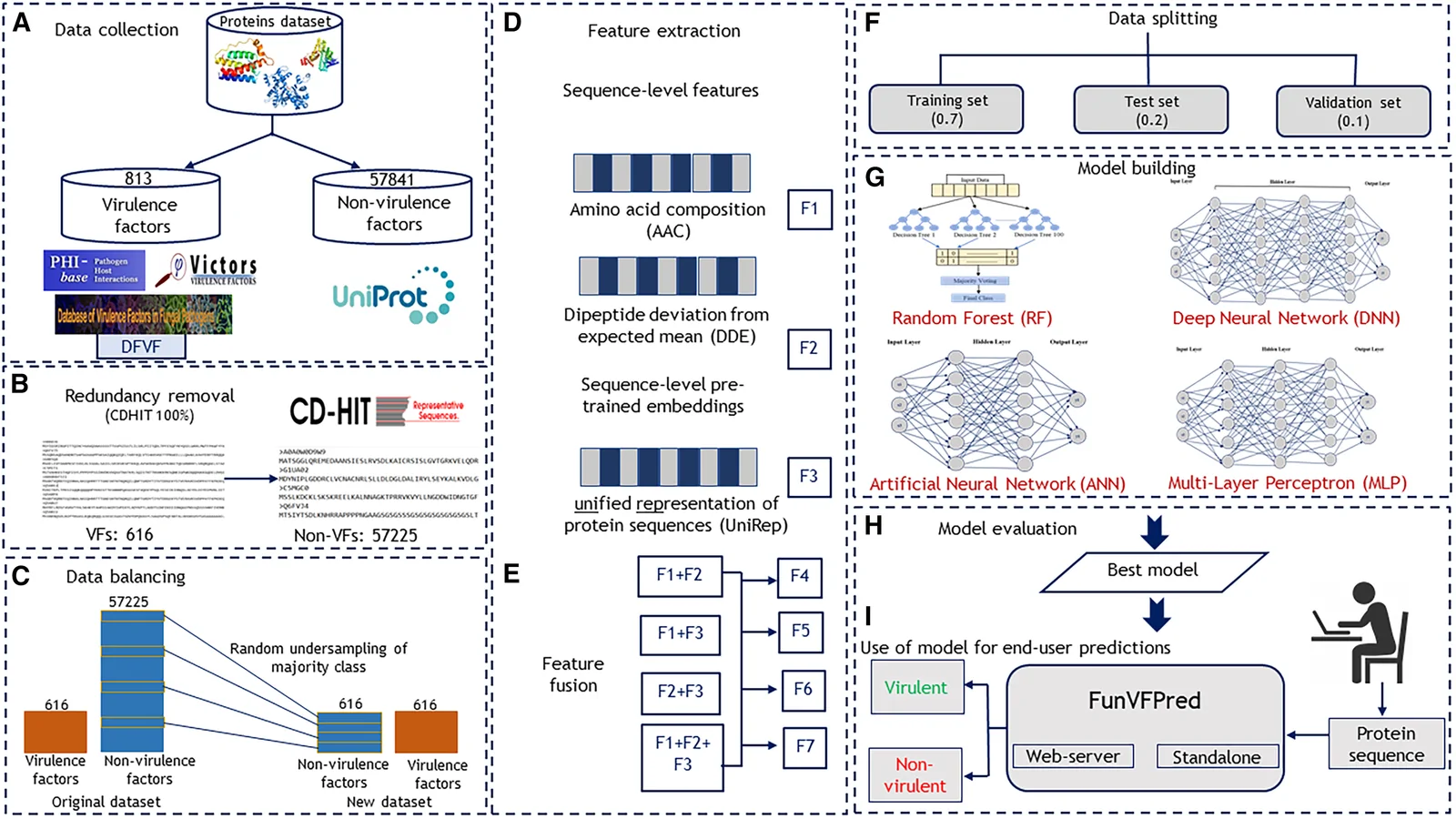
Schematic representation of the workflow of the proposed study (A–I) Pre-processing is shown in (A)–(E). (A) Data collection including positive (virulence factors) and negative data (non-virulence factors); (B) redundancy removal step; (C) the process of balancing the imbalanced data; (D) extraction of conventional features and sequence-based embeddings; (E) the feature fusion step; (F) the data splitting; (G) various classifiers to build model for binary classification; (H) evaluation of classifiers performance to identify the best predictive model; and (I) tool deployment for end-user prediction.

In this study, we present FunVFPred, an ML-based tool developed to predict VFs in human-pathogenic fungi. While existing tools such as EffectorP and FungalRV are tailored primarily to plant-pathogenic fungal effectors or specific classes of fungal proteins,^27^^,^^28^ FunVFPred aims to provide broader prediction of virulence-associated proteins relevant to human fungal infections. Because of the inherent biological differences between plant and human fungal pathogens, direct benchmarking of VFs in human fungal pathogens against tools trained primarily to predict plant-pathogen effectors may not provide a biologically equivalent comparison. For example, the datasets used to develop EffectorP and Fungtion primarily consist of secreted proteins associated with plant-pathogenic fungi.^27^^,^^31^ Consequently, these tools may not adequately represent the functional diversity and sequence complexity of VFs produced by human fungal pathogens. To address this challenge, FunVFPred utilizes a broad feature set derived from protein sequences to predict virulence-associated proteins across diverse functional classes. To achieve this, we developed a random forest (RF)-based approach trained on protein sequences from *Candida* species, integrating traditional sequence-based features—amino acid composition (AAC) and dipeptide deviation from expected (DDE) mean—with UniRep embeddings (Figure 1). UniRep, a deep representation learning model pre-trained on millions of protein sequences, captures rich sequence representations that encode structural and functional information in a 1900-dimensional embedding.^32^

We benchmarked the performance of the RF model against other ML and deep learning (DL) classifiers, including artificial neural networks (ANNs), multi-layer perceptrons (MLPs), and deep neural networks (DNNs). Among the evaluated models, the RF model consistently outperformed the other classifiers in distinguishing virulent from non-virulent fungal proteins. To evaluate its generalizability, FunVFPred was further validated on an independent test set containing sequences from *Aspergillus fumigatus*. By addressing the need for computational approaches to predict virulence-associated proteins in human fungal pathogens, FunVFPred provides a resource for fungal bioinformatics and may support the prioritization of candidate proteins for subsequent investigation, including potential antifungal drug and vaccine target discovery.

## Results

### Model building and classification results

The classification models were trained using different combinations of extracted features, including AAC, DDE, UniRep, and their fused feature sets (Table 1). The RF classifier, configured with 100 decision trees and a fixed random seed (42), demonstrated superior performance among the evaluated models. Using feature bagging and parallel processing, the RF model achieved an accuracy of 77.4% and the highest MCC of 0.5509 when trained on the fused AAC+DDE+UniRep feature set.

**Table 1:** Details of fused feature sets used for FunVFPred model development

Features name	Concatenate features	Final fused feature
AAC features + DDE feature	F1 + F2	F4
AAC features + UniRep embeddings	F1 + F3	F5
DDE features + UniRep embeddings	F2 + F3	F6
AAC features + DDE features + UniRep embeddings	F1 +F2 + F3	F7

To evaluate the predictive performance of the models, we assessed precision, recall, F-measure (F1-score), Matthews correlation coefficient (MCC), accuracy, and area under the curve (AUC) for each classification algorithm. Among the evaluated classifiers, RF achieved the highest F1-score and MCC, indicating strong predictive capability for distinguishing virulent from non-virulent proteins across different class sizes. Precision and recall analyses indicated a low false-positive rate, thereby reducing the likelihood of incorrectly classifying non-virulent proteins as virulent and minimizing unnecessary downstream experimental validation. Furthermore, comparison of individual and fused feature sets demonstrated that combining AAC, DDE, and UniRep features improved the sensitivity (recall) and specificity of virulence prediction, highlighting the potential value of integrating complementary compositional, sequential, and learned protein representations. Collectively, these evaluation metrics provide a comprehensive assessment of model performance and support the utility of FunVFPred for prioritizing candidate VFs in fungi.

The ANN model, composed of two hidden layers with 64 and 32 neurons, respectively, employed ReLU activation functions and dropout regularization (30% and 20%) to reduce overfitting. With a sigmoid activation function in the output layer, the ANN model achieved an accuracy of 75% when trained using the combined AAC+UniRep feature set.

The MLP model, incorporating three hidden layers with 64, 32, and 16 neurons, respectively, followed a similar activation strategy and achieved an accuracy of 75% when trained using the DDE+UniRep feature set.

The DNN model extended the MLP architecture by incorporating four fully connected layers, batch normalization, and progressively decreasing dropout rates. This configuration yielded an accuracy of 75.81% when trained using the AAC+UniRep feature set, highlighting the model’s ability to capture complex feature interactions. Despite its deeper architecture, the DNN model did not surpass the RF model in terms of overall accuracy and MCC.

**Figure 2:**
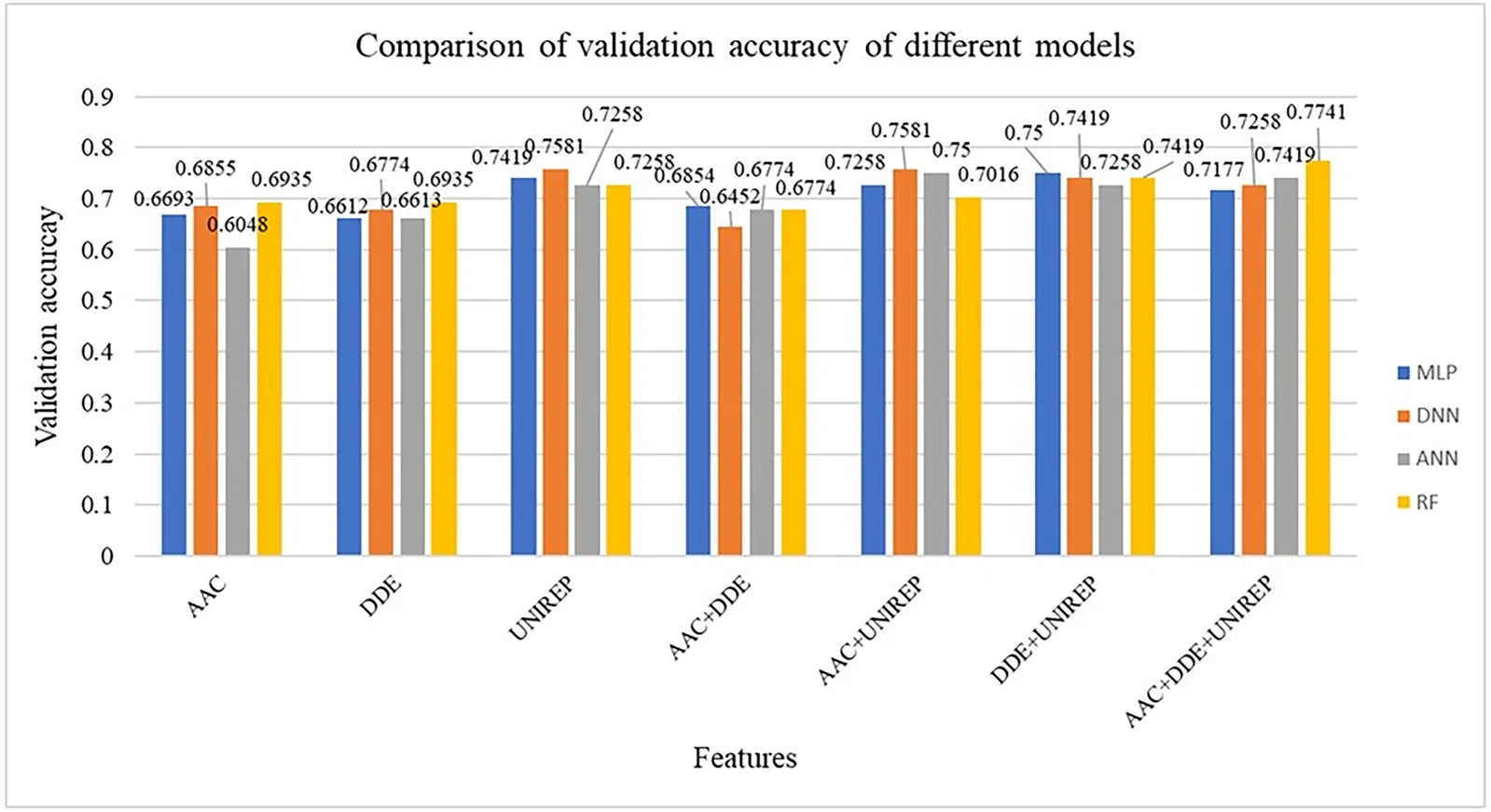
Comparison of accuracies of different machine learning and deep learning models This figure shows a comparison of validation accuracies across different machine learning and deep learning models used in the study. The models include random forest (RF), artificial neural network (ANN), multi-layer perceptron (MLP), and deep neural network (DNN). The comparison highlights how each model performed in terms of accuracy, helping to identify which approach was most effective for the prediction task.

**Figure 3:**
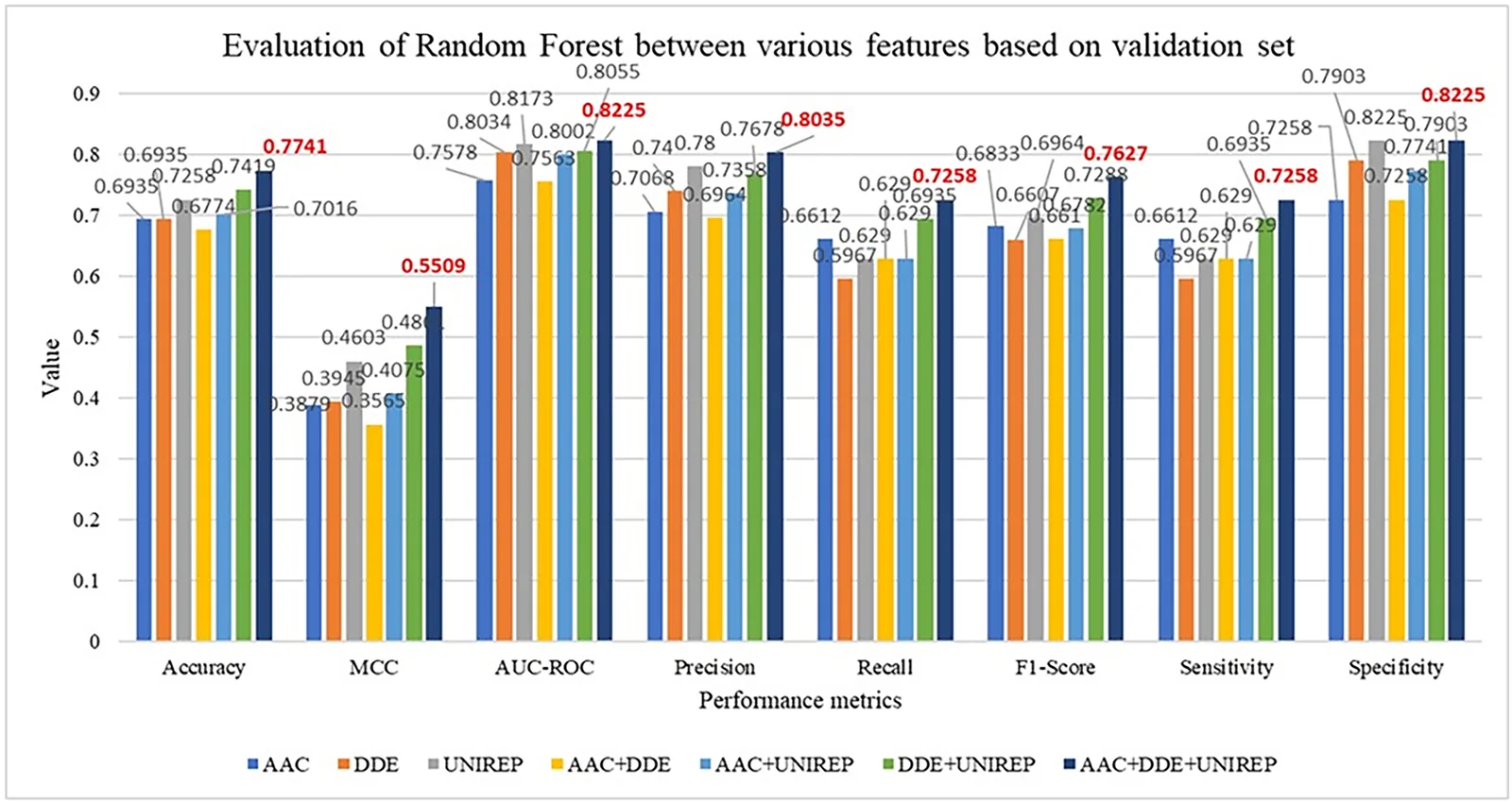
Performance metrics of the random forest classifier between different features The figure presents the performance metrics of the random forest (RF) classifier evaluated across different feature sets, including AAC, DDE, UniRep, and their merged combinations. The histogram illustrates key evaluation metrics such as accuracy, MCC, AUC-ROC, precision, recall, F1 score, sensitivity, and specificity. The comparison helps to assess how each feature type contributes to the model’s predictive performance.

Overall, the RF model consistently outperformed the other ML and DL classifiers evaluated in this study. It demonstrated robust performance in classifying virulent and non-virulent fungal proteins, achieving the highest accuracy of 77.4% (Figure 2; Table 2) and the highest MCC of 0.5509 (Figure 3; Table 2).

**Table 2:** Performance of different ML and DL models/classifiers with different features on the validation test set

Classifier	Features	Accuracy	MCC	AUC under the ROC curve
RF	AAC	0.6935	0.3879	0.7578
RF	DDE	0.6935	0.3945	0.8034
RF	UNIREP	0.7258	0.4603	0.8173
RF	AAC+DDE	0.6774	0.3565	0.7563
RF	AAC+UNIREP	0.7016	0.4075	0.8002
RF	DDE+UNIREP	0.7419	0.4861	0.8055
RF	AAC+DDE+UNIREP	0.7741	0.5509	0.8225
ANN	AAC	0.6048	0.2104	0.7310
ANN	DDE	0.6613	0.3241	0.7391
ANN	UNIREP	0.7258	0.4518	0.7994
ANN	AAC+DDE	0.6774	0.3595	0.7568
ANN	AAC+UNIREP	0.7500	0.5001	0.8301
ANN	DDE+UNIREP	0.7258	0.4526	0.8119
ANN	AAC+DDE+UNIREP	0.7419	0.4880	0.8439
MLP	AAC	0.6693	0.34	0.7393
MLP	DDE	0.6612	0.33	0.6992
MLP	UNIREP	0.7419	0.48	0.8251
MLP	AAC+DDE	0.6854	0.38	0.7319
MLP	AAC+UNIREP	0.7258	0.45	0.8344
MLP	DDE+UNIREP	0.7500	0.51	0.8069
MLP	AAC+DDE+UNIREP	0.7177	0.44	0.7950
DNN	AAC	0.6855	0.3722	0.7690
DNN	DDE	0.6371	0.2751	0.7106
DNN	UNIREP	0.7500	0.5001	0.8163
DNN	AAC+DDE	0.6452	0.2909	0.7447
DNN	AAC+UNIREP	0.7258	0.4518	0.8371
DNN	DDE+UNIREP	0.7177	0.4355	0.8040
DNN	AAC+DDE+UNIREP	0.7661	0.5357	0.8439

RF, random forest; ANN, artificial neural network; MLP, multi-layer perceptron; DNN, deep neural network; AAC, amino acid composition; DDE, dipeptide deviation from expected mean; UNIREP, unified representation.

### Feature importance analysis (RF contributions)

Of the top 50 features identified from the RF classifier trained on 2,320 features, 48 were UniRep embeddings, while the remaining two were DDE descriptors. This finding indicates that UniRep embeddings captured a substantial proportion of the predictive information encoded in the latent sequence representations. The two DDE descriptors may also capture biologically relevant sequence patterns associated with dipeptide distributions linked to virulence.

### Virulence-associated sequence pattern analysis

**Figure 4:**
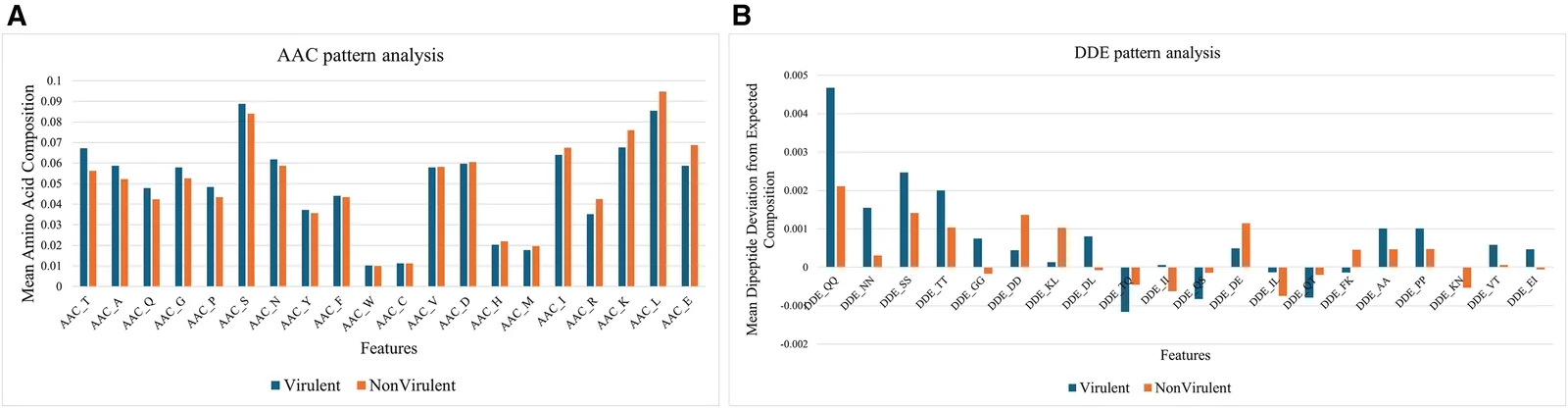
Characteristic sequence patterns associated with fungal virulence (A) Amino acid composition (AAC). Comparison of the relative frequencies of amino acids between virulent and non-virulent proteins. (B) Dipeptide deviation from expected (DDE) mean. Comparison of the relative frequencies of dipeptides between virulent and non-virulent proteins.

Analysis of AAC revealed an increased abundance of five amino acids (T, A, Q, G, and P) associated with virulence-related characteristics, including adhesion, biofilm formation, and host interactions (Figure 4A). DDE analysis identified recurring dipeptide motifs, including QQ, NN, SS, and TT, suggesting potential associations with virulence-associated protein characteristics (Figure 4B). Together, these analyses provide insights into sequence-level patterns that may contribute to the predictive performance of the selected model features.

### Performance across VF families

**Figure 5:**
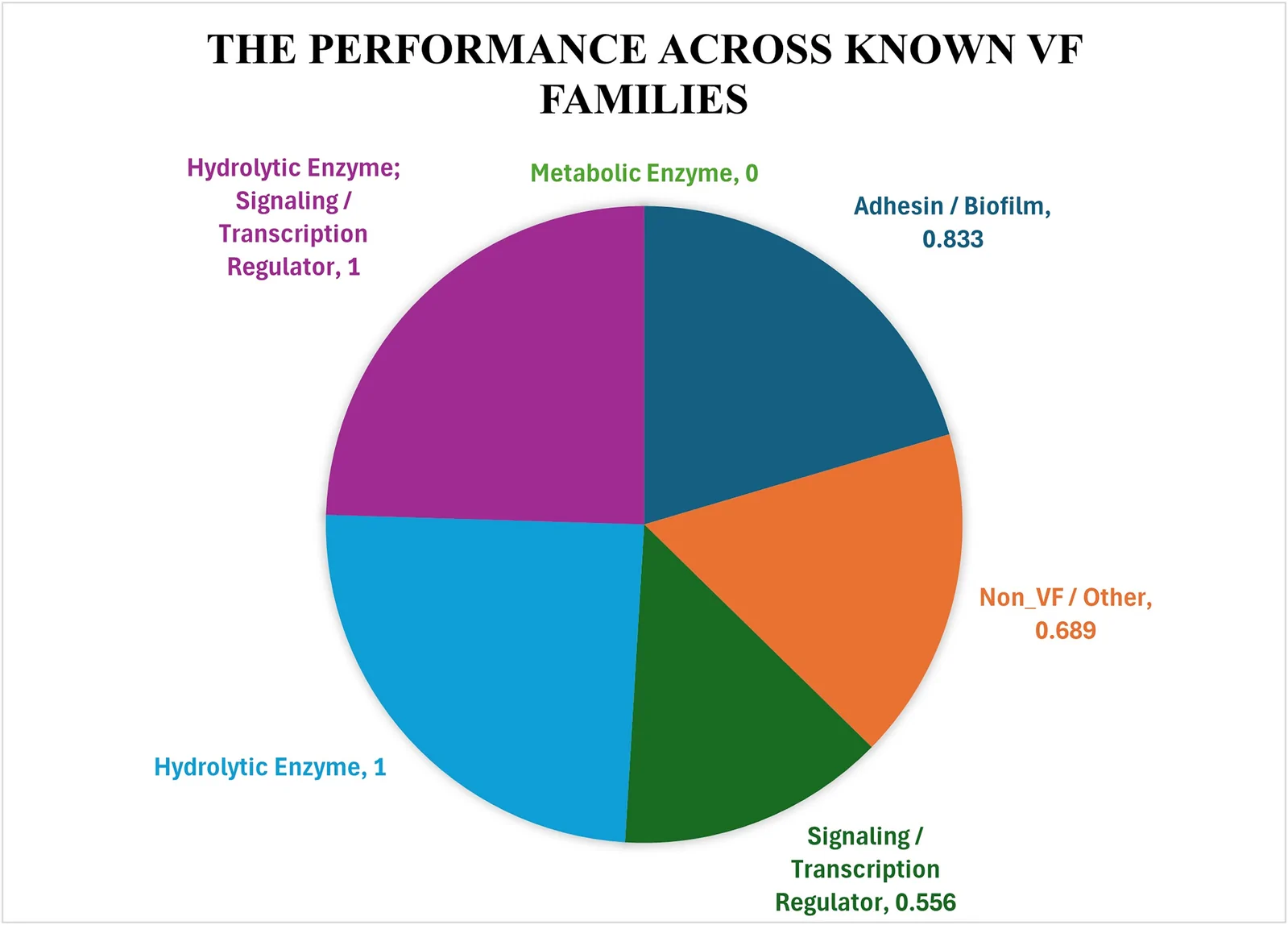
Distribution of proteins and prediction accuracy across virulence factor (VF) families The pie chart represents the proportion of proteins in each VF family, highlighting both virulent and non-virulent groups.

Using Gene Ontology annotations, predicted virulent proteins from different fungal species (Table 3) were grouped into functional families. Model sensitivity was high for adhesins/biofilm-associated proteins and hydrolytic enzymes, with accuracies exceeding 0.83. In contrast, metabolic and signaling regulators exhibited higher false-positive misclassification rates, potentially reflecting greater sequence heterogeneity within these functional groups (Table 4; Figure 5). These findings indicate that FunVFPred can identify sequence patterns associated with biologically relevant virulence mechanisms in human fungal pathogens, including those represented by experimentally characterized proteins such as ALS3, HWP2, BUD4, and VPS11.

**Table 3:** Fungal species and the corresponding number of virulent protein sequences included in the positive dataset after redundancy removal at 100% sequence identity

Fungal species	Number of virulent (positive) proteins	Number of virulent (positive) proteins after redundancy removal
*C. albicans*	692	508
*C. glabrata*	69	64
*C. tropicalis*	04	04
*C. parapsilosis*	18	15
*C. dubliniensis*	23	21
*C. orthopsilosis*	06	03
*C. glycerinogenes/Pichia kudriavzevii*	01	01
Total	813	616

**Table 4:** Performance of the random forest model across known VF families

VF family	Total proteins	Correct predictions	Incorrect predictions	Accuracy
Adhesin/Biofilm	18	15	3	0.833
Non_VF/Other	193	133	60	0.689
Signaling/transcription regulator	27	15	12	0.556
Hydrolytic enzyme	6	6	0	1.0
Hydrolytic enzyme; signaling/transcription regulator	1	1	0	1.0
Metabolic enzyme	1	0	1	0.0

The table summarizes the total number of proteins in each VF family, the number of correct and incorrect predictions by the model, and the corresponding prediction accuracy.

### RF performance on an independent dataset: Assessment of generalizability

The RF classifier outperformed the other evaluated ML and DL models (ANN, MLP, and DNN) on the validation dataset. To assess the broader applicability of FunVFPred, an independent dataset was constructed using sequences from fungal species that were not included in the RF training dataset. The model achieved an overall accuracy of 68.7% using the UniRep and DDE+UniRep feature sets, with an MCC of 0.4803, demonstrating the ability of FunVFPred to generalize to sequences from previously unseen fungal organisms (Table 5).

**Table 5:** Random forest prediction performance on the independent (blind) dataset

Features	Accuracy	MCC	AUC-ROC	Precision	Recall	F1-score	Sensitivity	Specificity
AAC	0.6875	0.4045	0.8437	0.6363	0.8750	0.7368	0.8750	0.5000
DDE	0.6250	0.2581	0.7265	0.6000	0.7500	0.6666	0.7500	0.5000
UNIREP	0.6875	0.4803	0.9140	0.6153	1.0000	0.7619	1.0000	0.3750
AAC+DDE	0.6875	0.3779	0.8046	0.6666	0.7500	0.7058	0.7500	0.6250
AAC+UNIREP	0.6250	0.2886	0.8437	0.5833	0.8750	0.7000	0.8750	0.3750
DDE+UNIREP	0.6875	0.4803	0.8203	0.6153	1.0000	0.7619	1.0000	0.3750
AAC+DDE+UNIREP	0.6250	0.3779	0.8125	0.5714	1.0000	0.7272	1.0000	0.2500

### RF prediction performance on validation and independent datasets using 5-fold cross-validation

**Figure 6:**
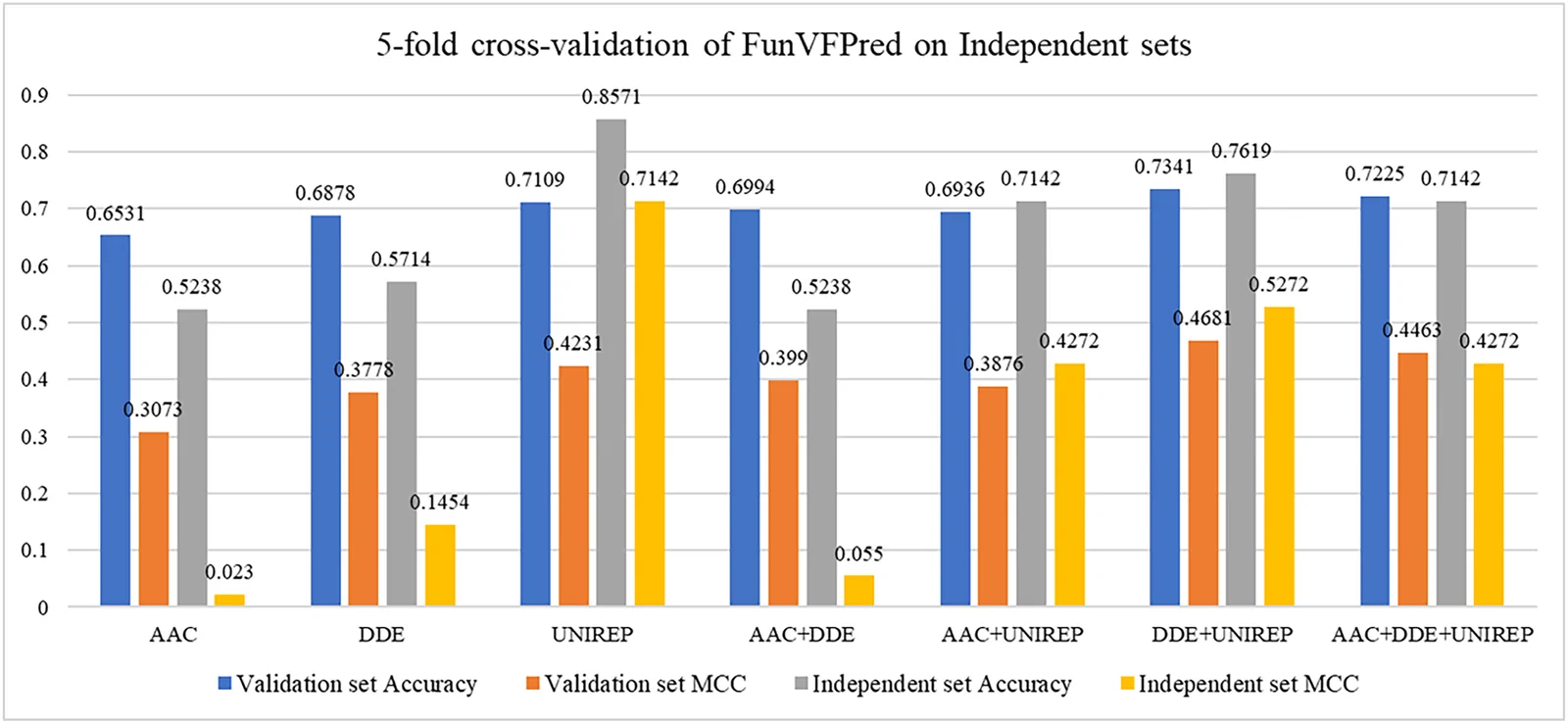
Performance of RF predictions for validation and independent datasets with 5-fold cross-validation The figure illustrates the performance of the FunVFPred tool using the random forest (RF) classifier, evaluated through 5-fold cross-validation. The results are shown for both the validation and independent sets, demonstrating the robustness and generalizability of the model’s predictions.

The RF model achieved high predictive performance using fused feature sets, with the AAC+DDE+UniRep combination yielding an accuracy of 77.4% and an MCC of 0.5509 for distinguishing virulent from non-virulent fungal proteins in the validation dataset. To further assess model robustness and generalizability, 5-fold cross-validation was performed on both the validation and independent datasets. The corresponding MCC values for the validation and independent datasets were 0.4681 and 0.7142, respectively. The accuracies for these datasets were 73.4% using DDE+UniRep and 85.7% using UniRep, respectively, as shown in Figure 6 and Tables 6 and 7. These results indicate that the RF classifier provides an effective approach for predicting fungal virulence-associated proteins. The incorporation of pre-trained protein embeddings such as UniRep contributed to improved predictive performance, with UniRep consistently enhancing results when used either independently or in combination with traditional sequence-derived features.

**Table 6:** 5-Fold cross validation of the random forest model on the validated set

Features	Accuracy	MCC	AUC-ROC	Precision	Recall	F1-score	Sensitivity	Specificity
AAC	0.6531	0.3073	0.7475	0.6413	0.6860	0.6629	0.6860	0.6206
DDE	0.6878	0.3778	0.7221	0.7105	0.6279	0.6666	0.6279	0.7471
UNIREP	0.7109	0.4231	0.8108	0.6956	0.7441	0.7191	0.7441	0.6781
AAC+DDE	0.6994	0.3990	0.7719	0.7073	0.6744	0.6904	0.6744	0.7241
AAC+UNIREP	0.6936	0.3876	0.7959	0.6853	0.7093	0.6971	0.7093	0.6781
DDE+UNIREP	0.7341	0.4681	0.8090	0.7325	0.7325	0.7325	0.7325	0.7356
AAC+DDE+UNIREP	0.7225	0.4463	0.8074	0.7065	0.7558	0.7303	0.7558	0.6896

**Table 7:** Random forest performance on the independent dataset using 5-fold cross-validation

Features	Accuracy	MCC	AUC-ROC	Precision	Recall	F1-score	Sensitivity	Specificity
AAC	0.5238	0.023	0.7772	0.5	0.2	0.2857	0.2	0.8181
DDE	0.5714	0.1454	0.6636	0.5454	0.6	0.5714	0.6	0.5454
UNIREP	0.8571	0.7142	0.8367	0.8571	0.8571	0.8571	0.8571	0.8571
AAC+DDE	0.5238	0.055	0.5090	0.5	0.6	0.5454	0.6	0.4545
AAC+UNIREP	0.7142	0.4272	0.7454	0.7	0.7	0.7	0.7	0.7272
DDE+UNIREP	0.7619	0.5272	0.7545	0.7272	0.8	0.7619	0.8	0.7272
AAC+DDE+UNIREP	0.7142	0.4272	0.7454	0.7	0.7	0.7	0.7	0.7272

Although virulence prediction tools such as VirulentPred, EffectorP, and FungalRV have demonstrated utility for predicting bacterial virulence proteins or fungal effectors, respectively, their specific training objectives and biological contexts differ from those of human fungal virulence prediction. Therefore, these tools were not included in direct performance comparisons. Instead, the statistically validated performance metrics of FunVFPred, obtained through 5-fold cross-validation and reported as mean ± standard deviation, provide an assessment of its predictive performance for virulence-associated proteins in human-pathogenic fungi.

## Discussion

There has been significant progress in understanding bacterial and viral VFs, but fungal VFs remain comparatively less characterized, particularly at the molecular and protein levels. To help address this gap, we developed and validated ML models specifically designed to predict VFs in human fungal pathogens. This approach aims not only to improve predictive performance but also to provide biologically meaningful insights that can guide future studies of fungal pathogenicity and antifungal strategies.

We evaluated four ML models: RF, ANN, MLP, and DNN, using a diverse set of protein features, including two conventional sequence-based features, namely, AAC and DDE, deep-learning-based UniRep embeddings, and combinations of these features through feature fusion. Our analysis showed that UniRep embeddings, both independently and when combined with traditional sequence-based features, improved model performance across validation and independent datasets. The improvement associated with UniRep suggests that learned protein sequence representations may capture information related to protein structure and function that is not fully represented by conventional sequence-derived features alone.

We employed a range of commonly used metrics, including accuracy (ACC), sensitivity (SN), specificity (SP), MCC, and area under the receiver operating characteristic curve (AUC-ROC), to evaluate classification performance. The binary classification framework provided a basis for applying ML algorithms to distinguish VFs from non-virulent proteins. Our analysis revealed that incorporating UniRep embeddings enhanced prediction performance. This improvement was observed when UniRep was used alone or in combination with traditional sequence-based features such as AAC and DDE, as demonstrated in Table 2. These results highlight RF as the most effective approach among the evaluated models, with the integration of pre-trained protein embeddings such as UniRep contributing to improved prediction performance. To further evaluate the RF classifier, its performance was assessed on an independent dataset.

### Performance evaluation of features used in the ML model

Traditional features, including dipeptide composition (DPC) and AAC, have been extensively applied in bacterial virulence prediction approaches such as PathoFact^33^ and VirulentPred.^16^ For example, PathoFact incorporated features such as AAC, DPC, and composition-, transition-, and distribution-based descriptors (CTDC, CTDT, and CTDD).^33^ Based on these established approaches, we incorporated commonly used sequence-derived features into our pipeline for predicting fungal VFs. However, our results showed that models using only conventional sequence-based features achieved moderate performance, with accuracies ranging from 62% to 68%. These results indicated that additional feature representations were required to improve prediction performance. We therefore explored UniRep embeddings, a pre-trained deep representation that captures 1900-dimensional protein sequence features derived from large-scale protein sequence datasets.

Our analysis identified UniRep, a unified representation for protein sequence embeddings, as a promising feature representation for protein classification. By integrating UniRep with conventional features into a single dataset, we improved the classification of virulent and non-virulent proteins using RF, which achieved an accuracy of 77.4% and an MCC of 0.5509 on the validation set. Testing on independent data demonstrated the ability of the model to generalize to previously unseen fungal sequences, achieving an accuracy of 68.7% and an MCC of 0.4803. UniRep is a sequence-based embedding approach trained on approximately 24 million UniRef50 protein sequences and generates a flexible representation of protein sequence properties that has been applied in protein informatics and protein engineering.^32^ Previous studies have also demonstrated the utility of UniRep representations. For example, Zhong et al. reported that a LightGBM model incorporating UniRep sequence embeddings together with secondary structure annotation (SSA) achieved the highest performance among the evaluated individual predictors.^34^

These observations prompted us to incorporate UniRep embeddings into our prediction pipeline, both independently and in combination with traditional features such as AAC and DDE. In 5-fold cross-validation, incorporation of UniRep substantially improved model performance, with accuracies exceeding 75% in the evaluated datasets. Furthermore, the use of UniRep embeddings resulted in consistently improved predictive performance across the validation and independent datasets, supporting their robustness as a protein representation for fungal VF prediction.

These findings indicate the potential of UniRep as a feature representation for virulence prediction. By leveraging deep representation learning, UniRep can encode complex sequence information that may not be adequately captured by conventional features alone. This may contribute to the ability of the model to distinguish between virulent and non-virulent proteins and supports the application of protein language model-derived representations in computational studies of fungal pathogenicity.

FunVFPred addresses an important computational need in fungal pathogenicity research by providing an approach specifically designed to predict fungal VFs. The predictive performance of the model was enhanced through the incorporation of UniRep embeddings, either independently or in combination with traditional sequence-based features such as AAC and DDE. Among the evaluated ML and DL approaches, RF demonstrated the strongest overall performance, achieving an accuracy of 77.4% and an MCC of 0.5509 on the validation set. Further evaluation on an independent dataset containing fungal sequences not included in the training data demonstrated the model’s ability to generalize, with an accuracy of 68.7% and an MCC of 0.4803. The robustness of the RF model was further assessed using 5-fold cross-validation, yielding an accuracy of 85.7% (MCC 0.7142) for the independent dataset and 73.4% (MCC 0.4681) for the validation dataset.

Beyond predictive performance, FunVFPred also provides opportunities for biological interpretation of predicted virulence-associated proteins. Several predicted proteins were associated with functional categories relevant to fungal pathogenicity, including adhesion, hydrolysis, and regulatory processes, which contribute to host colonization, tissue invasion, and evasion of host defense mechanisms. For example, in *Candida albicans*, proteins such as ALS3 and HWP2 play important roles in adhesion and biofilm formation, while BUD4 and VPS11 contribute to morphogenesis and pathogenicity. Comparison of predicted proteins with established classes of VFs provides additional support for the potential utility of FunVFPred in prioritizing candidates for experimental investigation and identifying potential targets for antifungal research.^22^

Feature importance and sequence pattern analyses further indicated that the predictions generated by FunVFPred can be examined in a biologically interpretable context. Adhesins and hydrolytic enzymes were predicted with relatively high accuracy, whereas predictions for signaling and metabolic proteins were comparatively less accurate. This difference may reflect the greater sequence heterogeneity of signaling and metabolic proteins compared with proteins involved in adhesion and hydrolytic functions. These observations suggest that future improvements in dataset size, annotation quality, and feature representation may further enhance VF prediction across diverse functional categories.

There are several limitations to our approach. First, publicly available databases may contain incomplete or unevenly distributed VF annotations, which can introduce biases during model training. Second, the predominantly *Candida*-based training dataset may limit model performance when predicting VFs from more distantly related fungal species. Third, as with many ML approaches involving high-dimensional learned representations, the use of deep protein embeddings may limit the biological interpretability of some model predictions. Despite these limitations, the developed framework provides a useful starting point for large-scale computational prioritization of candidate VFs in human-pathogenic fungi and for guiding subsequent experimental investigations.

FunVFPred integrates traditional sequence-based features with deep protein embeddings to provide a computational approach for predicting fungal virulence-associated proteins. The combination of biological interpretation and model-based prediction may facilitate the generation of hypotheses for experimental validation, prioritization of candidate antifungal targets, and further investigation of fungal virulence mechanisms. FunVFPred is freely accessible through its GitHub repository, GUI web server, and Docker container. Overall, FunVFPred provides a computational resource for prioritizing candidate virulence-associated proteins across fungal proteomes and supporting future studies of fungal pathogenicity.

### Limitations of the study

A key limitation of this study is the scarcity of experimentally validated fungal VF datasets. Unlike bacterial VFs, which benefit from large, well-curated repositories, fungal VF data in the public domain remain relatively scarce, limiting the diversity of virulence-associated proteins available for model development. In addition, although we compared our RF-based approach with multiple ML models, including ANN, DNN, and MLP, no dedicated computational tool is currently available for predicting fungal VFs in human-pathogenic fungi, precluding direct, tool-to-tool benchmarking. As additional experimentally validated fungal VF datasets become available, future studies may further improve model performance, enable more comprehensive benchmarking, and facilitate the development of increasingly robust computational approaches for fungal virulence prediction.

## Resource availability

### Lead contact

Further information and request for resources should be directed to the lead contact, Vishal Acharya (vishal.acharya@csir.res.in).

### Materials availability

This study did not generate new unique reagent or materials.

### Data and code availability

• The links for the dataset used in this study were publicly available and provided in the key resources table.
• The source code used in the study are available at GitHub link (https://github.com/ekjotkaurm/FunVFPred), GUI web server (http://14.139.59.213:8501/funvfpred/), and Docker container (https://github.com/ekjotkaurm/FunVFPred-GUI).
• Any additional information required to reanalyze the data reported in this paper is available from the lead contact upon reasonable request.

## Acknowledgments

The authors are thankful to 10.13039/501100001412Council of Scientific and Industrial Research (India) (10.13039/501100001412CSIR), India (CSIR in-house projects [MLP002619], iHUB &HCI Foundation grants [GAP-0338 and GAP0332], and Department of Biotechnology, India, under the aegis of HiCHiCoB Centre [GAP-0282] for infrastructural support). E.K. is thankful to 10.13039/501100001411Indian Council of Medical Research (10.13039/501100001411ICMR) for the Senior Research Fellowship award number (BMI/11(93)/2022). This manuscript represents 10.13039/100031822CSIR-IHBT communication number: 5805.

## Author contributions

E.K., conceptualization, methodology, visualization, and writing – original draft; V.A., conceptualization, methodology, visualization, writing – original draft, writing – review and editing, and supervision.

## Declaration of interests

The authors declare no competing interests.

## STAR★Methods

### Key resources table

**Table:** 

REAGENT or RESOURCE	SOURCE	IDENTIFIER
**Deposited data**		

Experimentally validated virulent proteins	PHI-Base	http://www.phi-base.org/
	Victors	http://www.phidias.us/victors/
	DFVF	http://sysbio.unl.edu/DFVF/
Non-virulent proteins	UniProt	https://www.uniprot.org/
Code availability	Present study (GitHub)	https://github.com/ekjotkaurm/FunVFPred
FunVFPred GUI	Present study	http://14.139.59.213:8501/funvfpred/
FunVFPred Docker	Present study	https://github.com/ekjotkaurm/FunVFPred-GUI

**Software and algorithms**		

SPAAN	Sachdeva et al., 2005	https://academic.oup.com/bioinformatics/article/21/4/483/202900
python3	Python	https://www.python.org/
numPy	NumPy	https://numpy.org/
pandas	Pandas	https://pandas.pydata.org/
sklearn	sklearn	https://scikit-learn.org/stable/
Random Forest	Random Forest	https://scikitlearn.org/stable/modules/generated/sklearn.ensemble.RandomForestClassifier/
Tensorflow	Tensorflow	https://www.tensorflow.org/
metaplotlib	Metaplotlib	https://matplotlib.org/
Torch	pytorch	https://pytorch.org/
biopython	biopython	https://biopython.org/
Joblib	joblib	https://joblib.readthedocs.io/en/latest/installing.html
tape-proteins	tape-proteins	https://pypi.org/project/tape-proteins/
Scipy	scipy	https://scipy.org/

### Experimental model and study participant details

No experimental model system used for this study.

### Method details

#### Data collection

For this investigation, three notable databases on fungal pathogens were used: the Database of Fungal Virulence Factors (DFVF),^15^ the Victors database,^35^ and the Pathogen–Host Interaction Database (PHI-Base).^36^ To ensure the quality and relevance of the collected data, stringent screening procedures were applied to the protein sequences extracted from these databases. Protein sequences experimentally validated as virulence factors (VFs) were collected from DFVF, Victors, and PHI-Base. These validations were based on functional evidence reported in the literature, including gene knockout experiments, infection models, or biochemical assays confirming pathogenic roles.

Initially, 813 experimentally validated pathogenic protein sequences or fungal virulence factors (VFs) were extracted from eight *Candida* spp., including *Candida albicans* (strain SC5314/ATCC MYA-2876), *C. glycerinogenes*, *Pichia kudriavzevii* (previously known as *Candida krusei*) (Taxon ID: 4909), *C. dubliniensis* (strain CD36/ATCC MYA-646/CBS 7987/NCPF 3949/NRRL Y-17841), *C. tropicalis* (Taxon ID: 5482), *C. parapsilosis* (Taxon ID: 5480), *C. orthopsilosis* (Taxon ID: 273371), *C. glabrata* (Taxon ID: 5478), and *C. metapsilosis* (Taxon ID: 273372). Redundancy removal was performed using CD-HIT,^37^ with a 100% sequence identity cutoff, resulting in 616 unique virulent proteins (Table 6).

The whole proteomes of these eight *Candida* spp. were downloaded from UniProt.^38^ Proteins overlapping with the positive dataset were excluded. The remaining proteins, which had not been experimentally characterized as virulent, were treated as putatively non-virulent. The final negative dataset comprised 57,841 proteins after applying a 100% sequence identity threshold and removing redundancy using the same CD-HIT approach.

This careful curation ensured that the positive dataset contained only experimentally validated virulent proteins, while the negative dataset was free from sequence overlap with the positive set. Minimizing redundancy within the two datasets resulted in high-quality input data for downstream feature extraction and predictive model development.

#### Data balancing

In real-world scenarios, there is a substantial imbalance between the number of virulent and non-virulent proteins, with non-virulent samples significantly outnumbering virulent proteins. Initially, our dataset contained 813 virulent and 57,841 non-virulent proteins. To investigate the impact of this imbalance on prediction performance, we created a reduced subset of the training data with a 1:10 ratio of virulent to non-virulent proteins. The same ratio was applied to the test and validation datasets.

This imbalanced subset was used to train the model and evaluate its performance. The results showed a strong bias toward predicting the majority (non-virulent) class, highlighting the importance of dataset balancing for accurate and fair classification.

To address this issue, we employed a random undersampling strategy to balance the classes at a 1:1 ratio. Specifically, non-virulent sequences were randomly sampled using a fixed seed to ensure reproducibility, and the final balanced dataset was prepared and saved using Biopython's SeqIO module. This approach ensured equal representation of both classes during model training, reducing prediction bias and improving classification balance.

#### Feature encoding

To enhance the prediction of VFs, we used sequence-based features, including Amino Acid Composition (AAC), Dipeptide Deviation from Expected Mean (DDE), and UniRep embeddings. UniRep is a pretrained deep learning model trained on a large corpus of protein sequences and is capable of capturing structural, evolutionary, and biophysical information from protein sequences.

The rationale for combining these features is both biological and technical: AAC captures overall amino acid usage and associated physicochemical properties, DDE captures local sequence dependencies that may be relevant to functional motifs, and UniRep embeddings provide high-dimensional learned representations of protein sequence properties. Collectively, these complementary features allow models to better capture the biological diversity of virulence-associated proteins.

#### Amino acid composition (AAC)

The frequencies of the 20 standard natural amino acids (i.e., “ACDEFGHIKLMNPQRSTVWY”) within a protein or peptide sequence are expressed as the amino acid composition (AAC) feature.^39^ The formula below was used to calculate AAC:

$$f\left(a\right)=\frac{N\left(a\right)}{N},a\in \left\{A,M,L,\ldots ..,P\right\}$$

where N(a) indicates the number of a particular amino acid that exist are, N is the protein or peptide's sequence length, and f(a) is the final 20-dimensional feature vector that was generated.

Protein sequences were read from a FASTA file using Biopython (version 1.81), and AAC values were calculated using NumPy for efficient computation. Each feature vector was then linked to its corresponding class label (virulent = 1, non-virulent = 0), as provided in a CSV file containing protein identifiers. The final dataset, combining AAC features and class labels, was saved in CSV format for use in ML models.

#### Dipeptide deviation from expected mean (DDE)

The DDE feature comprises three components: the theoretical mean (TM), dipeptide composition (DPC), and theoretical variance (TV).^40^ The TM characteristic was calculated as follows:

$$TM\;\left(a,b\right)=\frac{C_{a}}{C_{N}}\times \frac{C_{b}}{C_{N}}$$

where CN equals 61, which indicates the total number of potential codons excluding the three stop codons, and C_a_ and C_b_, respectively, indicate the codon numbers encoding amino acids a and b. The DPC feature calculation makes use of the above explanation. This is how the TV feature is computed:

$$TV\left(a,b\right)=\frac{TM\left(a,b\right)\left(1-TM9a,b\right))}{N-1}$$

Subsequently, the following formula is employed to calculate DDE(a, b):

$$DDE\left(a,b\right)=\frac{DPC\left(a,b\right)-TM\left(a,b\right)}{\sqrt{TV\left(a,b\right)}}$$

The DDE features were computed using an in-house Python script employing the Biopython package. Each protein sequence was parsed from a FASTA file, and its dipeptide composition was calculated. The generated features were merged with corresponding labels and saved in CSV format for downstream classification tasks.

#### Deep representation learning embeddings

UniRep^32^ is a deep representation learning method that uses a multidimensional Long Short-Term Memory (mLSTM) model with 1900 dimensions. It was trained on the UniRef50 protein database to learn complex features of amino acid sequences. High-dimensional embeddings that represent structural and functional characteristics of proteins are generated using this approach. UniRep provides a flexible representation of protein sequences and has demonstrated potential for applications in protein engineering and computational biology.

The UniRep feature calculation process can be summarized as follows:

1. Sequence encoding: Each amino acid in the protein sequence was represented using a 20-dimensional one-hot encoding vector.
2. Feature extraction using mLSTM: For each amino acid position t, a multiplicative long short-term memory (mLSTM) network processes the sequence to produce hidden states (h_t_).

$$h_{t}=mLSTM\left(x_{t,}\;h_{t-1},c_{t-1}\right)$$

where, *x*_*t*_ is input vector (one-hot encoding of amino acid at position *t*), *h*_*t*-1_ is previous hidden state and *c*_*t*-1_ is previous cell state

3. Sequence representation: For a protein sequence of length *T*, the final UniRep embedding can be either:
4. The last state (*h*_*T*_), or
5. The average of all hidden states (mean pooling):

$$UniRep=\frac{1}{T}\sum_{t=1}^{T}h_{t}$$

The UniRep features were extracted using the TAPE (Tasks Assessing Protein Embeddings) library in Python. Protein sequences were parsed from a FASTA file and encoded using a UniRep-compatible tokenizer. The pre-trained UniRep model (babbler-1900) was utilized to generate 1900-dimensional embeddings by averaging the hidden states across each sequence. The resulting feature vectors were combined with protein identifiers and corresponding labels from the input dataset. The final feature matrix was saved in CSV format for model development.

The selection of AAC, DDE, and UniRep features to represent important biological features associated with virulence proteins in fungi was made because these features provided complementary information regarding composition (AAC), sequence order (DDE), and evolutionary (UniRep) properties of the proteins of interest.

#### Feature fusion

To improve the predictive performance of ML and DL models, various feature representations were integrated. The amino acid composition (AAC) encodes the overall frequency of individual residues in a protein sequence, while the dipeptide deviation from expected mean (DDE) characterizes statistical deviations in dipeptide frequencies. In contrast, UniRep embeddings offer high-dimensional representations learned from large-scale protein data, capturing intricate evolutionary and contextual patterns. These distinct features provide complementary insights—ranging from basic compositional profiles and local sequence dependencies to deep, abstract representations.

By merging features from AAC, DDE, and pre-trained UniRep embeddings, feature fusion is accomplished as shown in Table 7. Concatenation serves to integrate these traits into a single, all-inclusive representation, improving their capacity to capture crucial biological data.

Feature integration was performed by aligning datasets on a shared identifier (protein_ids), ensuring accurate correspondence across records. Merging was carried out using Python's pandas library, followed by deduplication of label fields and standardization. Any missing entries introduced during the merge were imputed with zeros to maintain consistency across samples. This fusion of complementary representations enables models to leverage global composition, local sequence patterns, and deep learned structural or contextual information simultaneously, improving predictive performance. This approach enabled the construction of several comprehensive feature combinations as detailed in Table 7.

#### Data splitting

Following feature extraction and class balancing, the dataset was partitioned into three distinct subsets to facilitate model training, testing, and independent evaluation. Specifically, 70% of the data was allocated to the training set, which was used to develop ML and DL models. The test set, comprising 20% of the data, was used to evaluate model performance during development. An additional 10% was reserved as a validation set to assess the model's ability to generalize to unseen protein sequences, ensuring the robustness and reliability of the predictive framework.

To maintain equal representation of positive (virulent) and negative (non-virulent) classes, the dataset was stratified during the splitting process. Each class was separated and independently split using stratified sampling to preserve label distribution across subsets. After stratification, samples were randomly shuffled and recombined to form the final training, test, and validation sets. This careful partitioning strategy ensured that model evaluation remained unbiased while avoiding potential overfitting, thereby supporting reliable performance estimation.

Moreover, prior to dataset partitioning, duplication removal was performed to eliminate possibilities of bias in the similarities between additional sequences from the training, testing, and validation datasets.

#### Creation of an independent dataset for blind testing and generalizability assessment

In order to evaluate the prediction performance of our RF classifier, we prepared an independent dataset with sequences from a fungal species that was not included in the primary dataset (*Candida spp*.). PHI-Base, Victors, and DFVF were the same databases from which VFs from *Neosartorya fumigata* (*Aspergillus fumigatus*) were extracted as a positive, non-redundant dataset. This independent set, which is completely blind, was created exclusively to test the model's capacity to predict entirely new sequences. This enables us to evaluate the model's generalization ability to organisms that were not component of the training, test, or validation set. Random non-virulent protein sequences from the same fungal species were picked to create the negative dataset for independent set so as to match the 73 virulent proteins. The dataset was balanced to contain 73 virulent (positive) and 73 non-virulent (negative) sequences using a random undersampling technique. This dataset was subjected to the same feature extraction and preprocessing procedure and then split into three subsets: training (70%), testing (20%), and validation (10%). An impartial evaluation of the model's performance on the independent dataset was made possible by this comprehensive approach. Subsequently, five-fold cross-validation was implemented in conjunction with independent set to gauge the predictive ability of the RF model.

An independent dataset of data from other fungi, such as *Neosartorya fumigata*, not included in the main *Candida* dataset provided biological separation between the two datasets and decreased the chance of bias caused by a shared evolutionary history between the *Candida* and *Neosartorya* datasets.

#### Model building/classification

To evaluate the predictive ability of the extracted features, we trained classifiers using both deep learning (DL) and conventional machine learning (ML) algorithms. Specifically, we evaluated Deep Neural Networks (DNN), Multi-Layer Perceptrons (MLP), Random Forest (RF), and Artificial Neural Networks (ANN). The RF approach was selected because of its established ability to handle high-dimensional feature spaces while reducing the potential for overfitting through ensemble learning.^41^

The extracted features, including AAC, DDE, and UniRep, were used to train four different models (RF, ANN, MLP, and DNN), and their performances were compared to identify the most effective approach for distinguishing virulent from non-virulent fungal proteins. Given the limited availability of dedicated computational tools for predicting fungal virulence factors in human-pathogenic fungi, multiple ML and DL models were evaluated to assess their suitability for this task. Comparing DL architectures (MLP and DNN) with conventional ML approaches (RF and ANN) provided insight into the relative performance of these approaches for fungal VF classification.

The RF algorithm was implemented using 100 decision trees, bootstrap aggregation, and random feature selection at each split. Parallel computation was enabled across all available processor cores, and a random seed of 42 was used to ensure reproducibility.

The ANN model consisted of an input layer, two fully connected (FC) layers, and an output layer for VF prediction. To mitigate overfitting, the first FC layer included 64 neurons with ReLU activation and a 30% dropout rate. The second FC layer comprised 32 neurons with ReLU activation and a 20% dropout rate. The output layer contained a single neuron with a sigmoid activation function to predict probabilities for binary classification.

The MLP model for VF prediction consisted of three fully connected (FC) layers followed by an output layer. The first FC layer contained 64 neurons with ReLU activation, the second contained 32 neurons with ReLU activation, and the third contained 16 neurons with ReLU activation. The output layer contained a single neuron with a sigmoid activation function to predict probabilities for binary classification.

The DNN architecture used for VF prediction consisted of four fully connected (FC) layers followed by an output layer. To reduce overfitting and stabilize activations, the first FC layer contained 64 neurons with ReLU activation, batch normalization, and a 40% dropout rate. The second FC layer contained 32 neurons with ReLU activation, batch normalization, and a 20% dropout rate. Similarly, the third and fourth FC layers each contained 16 neurons with ReLU activation, batch normalization, and dropout rates of 10% and 20%, respectively. The output layer contained a single neuron with a sigmoid activation function to generate binary classification probabilities.

All models were trained using binary cross-entropy loss and optimized using the Adam optimizer. Hyperparameters were fine-tuned using the validation set, and the performance of each model was evaluated using the test set.

The architectures of the RF, ANN, MLP, and DNN models were designed to account for the high dimensionality of the feature space while maintaining a balance between model complexity and predictive performance. Hyperparameters, including the number of neurons, learning rate, batch size, and number of training epochs, were optimized using the validation set. To reduce overfitting, independent datasets for training, validation, and testing were used for model development and evaluation. In addition, comparative experiments were performed using individual and fused feature sets to assess the contribution of individual features to overall model performance and robustness.

#### Evaluation metrics

In addition to accuracy and AUC, additional metrics, including precision, recall (sensitivity), F1-score, and Matthews correlation coefficient (MCC), were used to provide complementary insights into model performance, particularly in the context of class imbalance. These metrics measure the correctness of positive predictions and the ability of the model to identify the minority class, represented by virulent proteins. False-positive predictions were also analyzed to assess potential downstream biological implications and to determine whether model predictions were consistent with biologically relevant patterns. MCC was included as a robust metric for evaluating binary classification performance, particularly for imbalanced datasets.

To thoroughly evaluate predictive performance, we used an independent test set and several standard evaluation metrics. These included recall or sensitivity (SN), specificity (SP), accuracy (ACC), F1-score, and Matthews correlation coefficient (MCC). The mathematical definitions of these metrics are given below:

$$SN=\frac{TP}{TP+FN}$$

$$SP=\frac{TN}{TN+FP}$$

$$ACC=\frac{TP+TN}{TP+FP+TN+FN}$$

$$F1\;score=2\times \frac{TP}{2TP+FP+FN}$$

$$MCC=\frac{\left(TP\times TN\right)-\left(FN\times FP\right)}{\sqrt{\left(TP+FN\right)\times \left(TN+FP\right)\times \left(TP+FP\right)\times \left(TN+FN\right)}}$$

Here, TP (true positives) represents the number of virulent proteins correctly predicted by the model, while TN (true negatives) refers to correctly predicted non-virulent proteins. FP (false positives) and FN (false negatives) denote incorrect predictions of non-virulent and virulent proteins, respectively.

Sensitivity refers to the model’s ability to correctly identify virulent proteins, and specificity measures its ability to correctly identify non-virulent proteins. Together with accuracy, F1 score, and MCC, these metrics provide a comprehensive and balanced understanding of the classifier's performance.

### Quantification and statistical analysis

The performance of all machine learning models (RF, ANN, MLP, and DNN) was evaluated using five-fold cross-validation. For each model, the mean ± standard deviation of the performance metrics across the five folds was calculated. Model performance was assessed using accuracy, precision, recall, F1-score, and Matthews correlation coefficient (MCC). Independent test datasets were further used to evaluate the generalizability of the best-performing model.

No hypothesis-testing statistical analyses (such as t-tests or ANOVA) were performed because the objective of this study was to assess predictive model performance rather than compare experimental groups. Therefore, statistical significance indicators (e.g., asterisks) were not applicable.

Although VirulentPred, EffectorP, and FungalRV are commonly cited prediction tools, they were developed for bacterial virulence proteins, fungal effectors, or adhesin prediction and are not specifically intended for predicting the broad spectrum of virulence factors in human fungal pathogens. As a result, direct benchmarking against these methods using our dataset was not considered biologically appropriate. Instead, the performance of FunVFPred was evaluated through cross-validation and independent validation using experimentally characterized fungal virulence proteins.
